# Limitations of the manual supination resistance test

**DOI:** 10.1186/1757-1146-8-S2-O2

**Published:** 2015-09-22

**Authors:** Paul J Bennett, Eleftheria Lentakis, Antonio Cuesta-Vargas

**Affiliations:** 1 School of Clinical Science, Queensland University of Technology, Brisbane, Qld, 4061, Australia; 2 The Podiatry Practice, Taylor Medical Centre, Woolloongabba, Qld, Brisbane, 4101, Australia; 3 Physiotherapy Department & Biomedical Research Institute of Malaga (IBIMA), Faculty of Health Sciences, University of Malaga, Spain

**Keywords:** clinical tests, gait, confounding, podiatry

## Background

The aim of this study was to asses results obtained from a range of commonly performed lower extremity “open and closed” chain kinetic tests used for predicting foot function and correlate these test findings to data obtained from the Zebris WinFDM-T system®. When performed correctly these tests are thought to be indicators of lower extremity function. Podiatrists frequently perform examinations of joint and muscle structures to understand biomechanical function; however the relationship between these routine tests and forces generated during the gait cycle are not always well understood. This can introduce a degree of variability in clinical interpretation which creates conjecture regarding the value of these tests.

## Methods

15 health subjects were recruited into this study. Subject's age, gender, activity levels and biometric data was recorded. A trained practitioner performed commonly utilised clinical assessments i.e, manual supination resistance test (MSRT), Jack's test, Lunge test, arch morphology analysis, fascia cord tension test and the hamstrings tension test [1-4]. Subjects planter foot pressure and force parameters were recorded on the Zebris WinFDM-T™ and the GAITrite™ walkway systems. SPSS (version 21 IBM) software was used to analyse the relationship between kinetic test results and key outcome measures. QUT ethics approval was obtained to conduct this research.

## Results

Of significance, variation in clinical interpretation may occur when assimilating results of open and close chain kinetic tests. Some interpretations appear confounded by variables such as angle and base of gait and body weight, particularly in the case of the manual supination resistance test (r= 0.661, p=0.007). When controlling for body weight, MSRT was not found to be predictive of differences in vertical ground reaction force during the gait cycle.

## Conclusions

Clinical assessment of theoretical “risk factors” proves challenging. While clinically meaningful relationships are thought to exist between biomechanical tests and computer aided gait assessment, the findings of this work call into question the clinical validity of key tests and care should be exercised when interpreting their findings.
